# Extirpation of a Sarcomatous Testicle

**Published:** 1831

**Authors:** Daniel L. Saunders

**Affiliations:** Perryville, Tenn.


Art. V. *Extirpation of a Sarcomatous Testicle.*—By Dr. Daniel L. Sanders, of Perrysville, Ten., communicated in a letter to Dr. Drake.

When I first performed this operation, (in the month of April, 30,) I was not impressed with the importance of reporting it, and therefore neglected it, until induced to do so by repeated solicitations from several medical gentlemen, who seemed to regard its publication, with a greater degree of interest.

The subject of the operation was a mulato man (Sam) aged forty-five years. The history which he gave me of his affliction, may be condensed into the following narrative:

About five years ago, he informed me, he discovered the first approach of this alarming enlargement of his testicle, under the form of a hydrocele; for about four or five months, its growth was rapidly progressive; at the expiration of which time the hydrocele was punctured, and the fluid evacuated. The coats of the scrotum were, however, shortly distended again by a deposition of the same fluid, which was again evacuated; and thus repeated many times. At length, the injection of some stimulating fluid was resolved on for the purpose of obliterating the cavity. Shortly after the injection of this fluid, my patient relates, the growth of the testicle commenced, which progressed gradually to such an enormous size, as to be greatly inconvenient to the patient. The operation was performed in the following manner:

The patient having been previously secured, after the manner usually practised in the operation of lithotomy, an incision was made, three inches in length, through the tunica albuginea, and tunica vaginolis, into the substance of the testicle. On examination, I found the coats completely coalesced, and finally attached to the testicle. They were also much indurated, and covered with blood vessels of an enormous magnitude; which, besides adding greatly to the already prodigious mass, much embarrassed the operation. The coats of the testicle were, however, slowly dissected from the testicle, while the larger vessels were secured by ligatures, as the hemorrhage seemed to demand, until the whole of the testicle was denuded up to the spermatic chord. Then carefully separating the vasdeperens, the cord was penetrated by a needle, armed with a double ligature, and securely tied, while the excision of the bulky mass completed the operation. The patient was put upon the course, usually resorted to in the treatment of wounds; and he remains, at this time, (twelve months from the time of the operation,) in good health.

Upon examination, the internal glandular structure of the testicle, seemed to be entirely wanting, or converted into a watery fluid, which, in form of a cyst, occupied the centre, while the cyst was enveloped by a sarcomatous mass, interspersed with scurbus indurations.

The testicle measured eight inches in length, and five in diameter, and weighed four pounds.

D. L. SAUNDERS.

DANIEL DRAKE, M. D.
*Memphis, Tennessee, 20th May,* 1831.

